# The “Top-Down” Approach to the Evaluation of Children with Febrile Urinary Tract Infection

**DOI:** 10.1155/2009/783409

**Published:** 2009-03-30

**Authors:** Hans G. Pohl, A. Barry Belman

**Affiliations:** Division of Urology, Children's National Medical Center, Washington, DC 20010, USA

## Abstract

The evaluation of children presenting with urinary tract infection (UTI) has long entailed sonography and cystography to identify all urological abnormalities that might contribute to morbidity. The identification of vesicoureteral reflux (VUR) has been of primary concern since retrospective studies from the 1930s to 1960s established a strong association between VUR, recurrent UTI, and renal cortical scarring. It has been proposed that all VUR carries a risk for renal scarring and, therefore, all VUR should be identified and treated. We will not discuss the controversies surrounding VUR treatment in this review focusing instead on a new paradigm for the evaluation of the child with UTI that is predicated on identifying those at risk for scarring who are most deserving of further evaluation by cystography.

## 1. Introduction

Concern for the influence of vesicoureteral reflux (VUR) on renal cortical scarring began with the recognition that VUR transmits bacteria to the upper urinary tract and the observation that APN, VUR, and renal scarring frequently coexisted in the same patient. Although APN was considered to be an ascending infection, it was mistakenly considered to always result from VUR [1, 2]. With these relationships established, the American Academy of Pediatrics Subcommittee on Urinary Tract Infection published a practice parameter that recommends renal-bladder sonography (RBUS) and voiding cystourethrography (VCUG) for the evaluation of urinary tract infection in febrile infants and young children [3].

Sonography is appealing as a method of evaluation following UTI; however, its ability to detect upper urinary tract abnormalities is limited. Although RBUS is noninvasive and sufficiently sensitive to evaluate collecting system dilatation, controlled trials have found it to be less sensitive than contrast cystography and 99m-Tc-dimercaptosuccinic acid (DMSA) renal scintigraphy in detecting VUR or parenchymal lesions. As much as 60% of reflux and 50% of renal scan abnormalities noted on DMSA are routinely missed by sonography [4–6]. Given the prevalence of prenatal sonography, few RBUSs performed for the evaluation of febrile UTI demonstrate clinically significant abnormalities [7]. Alternatively, VCUG is the gold standard for the detection of VUR; however, it is invasive, requiring catheterization, generally of the nonsedated child. Despite the liabilities of this evaluation paradigm, it remains the recommended guideline, although there is a tendency for children not to undergo recommended testing, particularly when treated as outpatients for their UTI [8]. These limitations combined with an increasing clinical experience with DMSA renal scans has led some to advocate a novel paradigm for the evaluation of children with UTI, termed the “top-down approach” which focuses on identifying children at risk for renal scarring, whether or not VUR is present, as compared with the recommended scheme which proposes to identify all VUR. Both approaches employ ionizing radiation to image the urinary tract, but a direct comparison of effective doses cannot be made reliably because the radiation is focused in the one case (cystography) and diffused throughout the body in the other (DMSA renal scan). Gonadal dosimetry is lower for continuous fluoroscopic cystography (effective dose, 0.45 mSv) as compared with DMSA renal scans (effective dose, 1.8 mSV; ovary, 85 mrads; testis 45 mrads) but as we will demonstrate, the “top-down” approach offers significant advantages that outweigh this potential liability.

## 2. Renal Scintigraphy in the Evaluation of UTI, VUR, and Acquired Renal Cortical Abnormalities

Experimental studies in the refluxing piglet model of ascending acute pyelonephritis were conducted using strict histopathologic criteria as the standard of reference to evaluate the true sensitivity and specificity of renal cortical scintigraphy [9, 10]. These experimental studies, as well as clinical observations, have facilitated our ability to discriminate between lesions consistent with acute inflammation and those that represent congenital or acquired abnormalities of the renal cortex. Acute inflammation results in relative photopenia of the renal cortex without loss of the renal contour. Over time, inflammation can lead to scarring of the renal cortex that produces loss of volume as renal tissue contracts. This volume loss is represented as disruption of the renal contour with photopenia. Renographically, congenital abnormalities of the renal cortex can look similar to those that are acquired following infection, the ability to distinguish between the two depends on whether the child had no UTI prior to the abnormality being discovered (congenital) or whether a DMSA scan previously demonstrated no cortical lesion or only those consistent with inflammation (acquired). In the absence of acute inflammation, the presence of congenital abnormalities of the renal cortex, such as hypoplasia or volume loss, suggests VUR and warrants cystography. Identification of developmental renal parenchymal abnormalities is most accurately made in the newborn in whom one knows UTI did not play a role. Beyond that, it may be difficult to differentiate acquired from congenital defects regardless of UTI history.

The DMSA scan was determined to be highly sensitive and reliable for the detection and localization of experimental acute pyelonephritis, with a sensitivity of 87 to 89 percent and specificity of 100 percent for both. When individual pyelonephritic lesions were analyzed, DMSA scan findings correlated with histopathological changes with an overall agreement rate of 89 to 94 percent. Those lesions not detected were microscopic foci of inflammation not evident on gross examination and not associated with significant parenchymal damage. DMSA nuclear renography has been shown to be the most accurate (96%) for the detection of APN as compared with gadolinium-enhanced MRI (91%), contrast CT scan (90%), and power Doppler sonography (69%) [11].

The clinical diagnosis of acute pyelonephritis traditionally has been made on the basis of the classic signs and symptoms of fever and flank pain or tenderness associated with pyuria and positive urine culture. However, accurate diagnosis based solely on these parameters is often incorrect, particularly in neonates and infants [12]. Despite the fact that the majority of patients (50 to 80 percent) with fever and systemic clinical findings consistent with acute pyelonephritis have abnormal DMSA scan findings, there is still a high false-positive and/or negative rate based on routine clinical and laboratory parameters, including fever, elevated WBC, elevated c-reactive protein (CRP), elevated erythrocyte sedimentation rate (ESR), and the presence of VUR [13]. Not only is DMSA scintigraphy highly sensitive and specific for the diagnosis of acute pyelonephritis, but it also provides important information regarding renal function and the extent of renal parenchymal inflammation. Documentation of renal parenchymal damage associated with acute pyelonephritis is fundamental to understanding the roles of infection and vesicoureteral reflux in the etiology of pyelonephritis and renal scarring.

The critical role that infection plays in the pathogenesis of renal scarring associated with VUR was clarified by Ransley and Risdon's experimental studies on the refluxing piglet model, by which—in the face of VUR and normal voiding pressures—they showed that renal scarring occurs only when UTI is present [14]. While a single episode of APN can lead to significant renal damage, a clear association between the number of APN attacks and the incidence of renal scarring has been reported [15–19]. Renal scarring can be prevented or diminished, whether or not APN occurred in the face of VUR, by the appropriate administration of antibiotics [15, 20–22]. Based on all of the above, it is clear that infection, not reflux alone, is a prerequisite for acquired renal scarring.

## 3. Evolution of Acute Inflammation to Postinfection Scars

Several investigators have now evaluated the evolution of the acute inflammatory changes associated with pyelonephritis using serial DMSA renal scans [23–27]. Acute DMSA renal scan defects persisted as renal scars in 36 to 52 percent of kidneys. The sites of new renal scarring corresponded exactly to those sites of acute pyelonephritis seen on the initial DMSA renal scans, confirming the primary role of the acute inflammatory response to infection in the etiology of acquired renal scarring. Contralateral normal kidneys and initially uninvolved areas of abnormal kidneys have almost always remained normal on follow-up DMSA renal scans.Surprisingly, reflux has been identified in only 25 to 50 percent of kidneys that developed new renal scarring. This is attributable in part to the fact that the majority of patients (63 to 75 percent) with acute inflammatory changes on the initial DMSA renal scans did not have VUR. In one prospective study of 38 kidneys with initially abnormal DMSA scans, scarring developed in 6 of 15 (40 percent) kidneys with associated VUR and in 10 of 23 (42 percent) kidneys without demonstrable reflux [25]. Others have observed similar findings [28]. These observations provide convincing clinical evidence that renal parenchymal infection, rather than vesicoureteral reflux, is the prerequisite for acquired (postnatal) renal scarring and that the incidence of VUR in children with acute pyelonephritis is lower than commonly appreciated.

Despite these findings, the importance of VUR (particularly grades III or higher) as a risk factor for renal scarring should not be discounted. Clearly, patients with moderate and severe reflux are much more likely to develop acute pyelonephritic damage than children with mild or no reflux [12, 29]. Furthermore, although 62 percent of the kidneys with postpyelonephritic renal scarring in one study were drained by nonrefluxing ureters, renal scarring was still significantly more common in those kidneys with grade III or higher VUR compared with kidneys with mild or no reflux [29]. Thus, the increased propensity for scarring in patients with higher grades of VUR is *attributable* in part to the increased risk of these kidneys for acute inflammatory damage at the time of the initial infection [12, 28, 30].

## 4. The “Top-Down” Approach

The DMSA renal scan could supplant the standard evaluation scheme since it can better discriminate which child is at risk for renal scarring irrespective of the presence of VUR. In this novel algorithm, a child who presents with clinical signs and symptoms suggestive of pyelonephritis first undergoes a DMSA renal scan to detect renal inflammation. Once radiographic evidence of renal involvement is demonstrated, the child then undergoes cystography to search for VUR. This approach is supported by the following two studies.

Hansson et al. retrospectively reviewed 303 children under 2 years of age who presented with a first UTI (82% of whom had fever on presentation) [31]. VUR was found in 26% of the children (80/303), 66% of whom had abnormal DMSA scans, while no abnormalities were detected in the remaining 27 patients. An approach based on identifying renal cortical abnormalities prior to obtaining a VCUG would have identified not only the 66% of children with VUR presumably at risk for further scarring, but it would also have identified the 46% of the children without VUR who were also at renal risk, and most importantly, it would have excluded 120 children (40%) without VUR from a VCUG. One obvious criticism might be this approach inability to detect 34% (27/80) of VUR without renal involvement. However, 74% of these were low grade while the remaining seven cases of VUR—all grade III—either resolved or improved significantly during a two-year period follow-up period with no episodes of recurrent UTI and no new renal scars documented.

In a follow-up study, Preda et al. prospectively evaluated 290 children less than 1 year of age with UTI (79% of whom had fever on presentation) with VCUG and DMSA renal scans [32]. Fifty-one percent of the patients had positive scans, including 85% (44/52) of the children later found to have VUR. Among the eight cases of VUR that were “missed” by DMSA scans, 7 were of low grade, the remaining boy has grade III VUR with no acquired renal scars in follow-up despite an episode of breakthrough UTI.

This algorithm is further supported by the observations that while clinically significant acute lesions may occur in the presence or absence of VUR, significant renal scarring is most likely to occur in those with grades III and IV VUR [33]. Taken together, these studies suggest that the “top-down approach” is able to identify all children at risk for upper tract damage following UTI, whether or not they have VUR, and excludes children who may not be at risk, again irrespective of the presence of VUR. The sensitivity of this approach is improved by performing the DMSA renal scan as close to the time of the febrile UTI as possible. In the absence of acute inflammatory changes, the presence of congenital abnormalities of the renal cortex such as hypoplasia or focal volume loss typical of scarring is highly suggestive of VUR and warrants cystography.

## 5. Cost Savings of the “Top-Down” Approach

The “top-down” approach also serves to reduce the overall charges for the evaluation of children with UTIs. How great a reduction depends on what estimates are used for the prevalence of patients with VUR among children with febrile UTI, and among those with positive DMSA renal scans as well as the timing of evaluation. DMSA scans performed at a longer interval following the acute episode are less likely to demonstrate pyelonephritis, as many as half of those lesions having resolved. In Table 1, we present charge data for Children's National Medical Center and the assumptions used to calculate the cost per diagnosis of VUR by the traditional “bottom-up” approach ($3368–$8420) versus the “top-down” approach ($2630–$3033). This should not be surprising since almost half of the charge associated with the standard “bottom-up” approach is associated with sonography, a study that rarely identifies significant abnormalities in children with UTIs in contemporary series. If one routinely obtains DMSA renal scans on children with moderate to severe VUR, then the cost of this additional examination would further increase the charges associated with the bottom up approach in up to 25% of the children with VUR.

## 6. Recommendations

In our effort to identify those children who are most at risk, we might be tempted to apply certain demographic facts to further minimize studies. Patient age is recognized as being a risk factor for renal scarring; younger individuals are thought to be more susceptible. Gender may also play a role as younger males, particularly those who are not circumcised, tend to be at greater risk for UTI as well as high grade reflux. Reflux in blacks is also less common; however the renal effects are the same as in other groups in those who have reflux [34]. Finally, older children may be less susceptible to renal scarring following pyelonephritis.

Therefore, a negative evaluation by any means, whether by the traditional approach or by the “top-down” approach, may not be sufficient to exclude all children at risk. Therefore, those who present with recurrent febrile UTI deserve further attention. We, therefore, recommend that a prudent approach includes DMSA renal scans for all children with febrile UTI, ideally as close to the acute episode as possible. Those with positive scans should undergo cystography. If VUR is identified, the clinician should then formulate an appropriate treatment plan depending on various factors such as likelihood of VUR resolution, likelihood of renal scarring, taking into consideration parental concerns regarding various treatments, and their outcomes. Children with a negative DMSA scan require no further evaluation unless recurrent febrile UTI occurs, in which case cystography should be performed. The use of antimicrobial prophylaxis should be recommended based on known risks for recurrent UTI and for renal scarring should UTI occur regardless of the presence of absence of reflux.

## 7. Summary

The “top-down” evaluation provides an assessment of risk for renal scarring that strongly correlates with the presence of clinically relevant VUR. In addition to being a more cost-effective means of evaluating children with febrile UTIs, it is also predicted to lessen medical care costs by reducing the number of children on antibiotic prophylaxis and of children undergoing surgery for reflux that is unlikely to result in renal scarring.

## Figures and Tables

**Table 1:** 

	CPT code	Charge	Total charge
U/S, renal bladder	76770*	$651	$832
	76770.26*	$181	
VCUG	74455	$779	$852
	74455.26	$73	
DMSA	78700	$781	$968
	78700.26	$187	

*Assumptions used in calculations*:			
Positive DMSA renal scans among children with febrile UTI			50–80%
Presence of VUR among children with febrile UTI			25–50%
Presence of VUR among children with positive DMSA scan			66%

*Charge per case of VUR diagnosed*			
Bottom-up			$3368–$8420
Top-down			$2630–$3033

*The first CPT code and charge relates to the technical fee for performing the examination, while the second code (.26 modified) relates to the professional component.
